# A prospective observational study of Gallium-68 ventilation and perfusion PET/CT during and after radiotherapy in patients with non-small cell lung cancer

**DOI:** 10.1186/1471-2407-14-740

**Published:** 2014-10-02

**Authors:** Shankar Siva, Jason Callahan, Tomas Kron, Olga A Martin, Michael P MacManus, David L Ball, Rodney J Hicks, Michael S Hofman

**Affiliations:** Division of Radiation Oncology and Cancer Imaging, St Andrews Place, East Melbourne, 3002 Australia; Sir Peter MacCallum Department of Oncology, The University of Melbourne, Parkville, 8006 Australia; Department of Medicine, The University of Melbourne, Parkville, 8006 Australia

**Keywords:** Positron emission tomography, Definitive radiation, Lung cancer, 4D, Adaptive radiotherapy, Biological dose escalation, Biomarkers, Gamma-H2AX, Inflammatory cytokines

## Abstract

**Background:**

Non-small cell lung cancer (NSCLC) accounts for 85% of lung cancers, and is the leading cause of cancer deaths. Radiation therapy (RT), alone or in combination with chemotherapy, is the standard of care for curative intent treatment of patients with locally advanced or inoperable NSCLC. The ability to intensify treatment to achieve a better chance for cure is limited by the risk of injury to the surrounding lung.

**Methods/Design:**

This is a prospective observational study of 60 patients with NSCLC receiving curative intent RT. Independent human ethics board approval was received from the Peter MacCallum Cancer Centre ethics committee. In this research, Galligas and Gallium-68 macroaggregated albumin (MAA) positron emission tomography (PET) imaging will be used to measure ventilation (V) and perfusion (Q) in the lungs. This is combined with computed tomography (CT) and both performed with a four dimensional (4D) technique that tracks respiratory motion. This state-of-the-art scan has superior resolution, accuracy and quantitative ability than previous techniques. The primary objective of this research is to observe changes in ventilation and perfusion secondary to RT as measured by 4D V/Q PET/CT. Additionally, we plan to model personalised RT plans based on an individual’s lung capacity. Increasing radiation delivery through areas of poorly functioning lung may enable delivery of larger, more effective doses to tumours without increasing toxicity. By performing a second 4D V/Q PET/CT scan during treatment, we plan to simulate biologically adapted RT depending on the individual’s accumulated radiation injury. Tertiary aims of the study are assess the prognostic significance of a novel combination of clinical, imaging and serum biomarkers in predicting for the risk of lung toxicity. These biomarkers include spirometry, 18 F-Fluorodeoxyglucose PET/CT, gamma-H2AX signals in hair and lymphocytes, as well as assessment of blood cytokines.

**Discussion:**

By correlating these biomarkers to toxicity outcomes, we aim to identify those patients early who will not tolerate RT intensification during treatment. This research is an essential step leading towards the design of future biologically adapted radiotherapy strategies to mitigate the risk of lung injury during dose escalation for patients with locally advanced lung cancer.

**Trials registration:**

Universal Trial Number (UTN) U1111-1138-4421.

## Background

Local treatment failures are still a major cause for the disappointing outcomes for patients with non-small cell lung cancer (NSCLC) treated with radiotherapy. Locoregional failures still occur in up to 37% of patients [1], and is a major cause of the morbidity and mortality related to this disease. To minimise the risk of failure, a focus of current international research is radiotherapy dose intensification. Efforts to intensify radiotherapy are severely limited by the need to constrain dose to the surrounding normal lung in order to preserve function [2]. Unfortunately, acute lung injury secondary to RT in the form of pneumonitis is a potentially debilitating toxicity, sometimes leading to patient death. A recent meta-analysis suggests that symptomatic pneumonitis still occurs in 29.8% of patients and fatal pneumonitis in 1.9% [3]. However, currently used RT planning constraints that are designed to limit the risk of pneumonitis are based on evidence over a decade old [4]. These constraints are based on population-based volumetric measurements of total irradiated lung irrespective of regional variation of function, and do not account for individual variation in pulmonary physiology. Recent efforts to non-adaptively dose-escalate without personalizing radiotherapy to the individual’s risk of pneumonitis have met with limited or no success [5]. On the other hand, it has been estimated that tumour control probability (TCP, or likelihood of cure) for conventional radiotherapy could be improved by ~50% (from 19.9% to 28.7%) by adaptively intensifying radiotherapy [6]. At present, an understanding of the relationship between toxicity, radiation dose and volume of irradiated lung is incomplete. Acquiring normal human lung tissue after irradiation for pathobiological analysis is associated with significant patient risk. It is therefore imperative to establish *in vivo* functional imaging biomarkers for early assessment, prediction and ultimately avoidance of delayed organ dysfunction.

### In-vivo biomarkers of radiation effect in lung

Clinical, radiographic, and lung function endpoints have all been previously used to investigate the effects of inhomogenous irradiation of partial lung volumes [7]. Pulmonary function tests (PFTs) are tools capable of assessing global lung function as a whole organ. Reductions in pulmonary function have been used as an objective assessment of radiation-induced lung injury by several groups [8–10]. In the setting of breast cancer and lymphoma, Theuws et al. [11] postulated a 1% reduction in PFT for each 1-Gy increase in mean lung dose. Gergel et al. [12] investigated radiation-induced lung changes after irradiation of oesophageal cancers. This group found a statistically significant correlation between the volume of lung receiving between 7 – 10 Gy and reductions in total lung capacity, vital capacity, and carbon monoxide diffusion capacity. However, in the case of centrally located lung tumours, PFTs may improve post-irradiation due to reinflation of lung segments obstructed and collapsed by tumour. This has been previously reported in up to 40-50% of patients with centrally located tumours [13, 14].

Ventilation and perfusion (V/Q) imaging is an *in-vivo* technique that measures regional lung function and may be used to individualise lung radiotherapy. Assessment of lung perfusion (Q) is particularly relevant to radiation-induced lung damage as, along with pneumocytes, vascular endothelium is considered one of the most radiation sensitive tissue in the lungs [15]. Planar scintigraphy using ^99m^Tc-labeled macroaggregated albumin (MAA) is a long-established imaging standard for functional lung perfusion evaluation. Single positron emission computed tomography (SPECT) is a more modern functional assessment technique enabling three dimensional imaging [16], which has lead to improved sensitivity, specificity, and reproducibility [17–19]. The advent of hybrid SPECT/CT devices further improved diagnostic accuracy by enabling anatomic characterization of scintigraphic abnormalities [20]. Perfusion SPECT/CT has been demonstrated to improve functional lung avoidance during lung radiotherapy planning by several groups [21–23].

### PET/CT for ventilation and perfusion imaging

**Figure 1:**
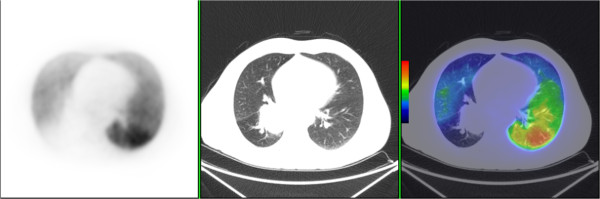
**MAA-perfusion PET (left), contemporaneous CT (middle), and co-registered perfusion PET/CT, in a patient with a right upper lobe T3 squamous cell carcinoma.**

**Figure 2:**
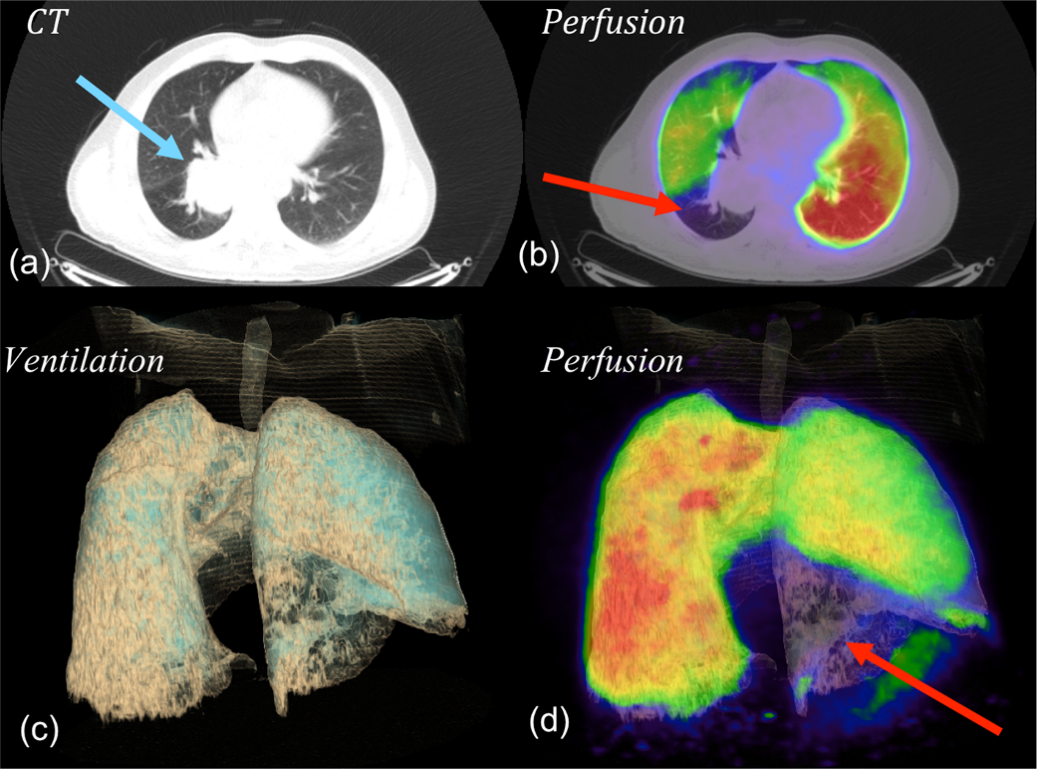
**V/Q PET/CT, a) CT alone, b) fused PET/CT, c) 3D volume rendered (VR) CT ventilation reconstruction d) 3D fused VR perfusion PET/CT. A patient with a large upper lobe NSCLC (** ***image a*** **), showing both ventilation deficits distal to tumour and perfusion deficits distal to the tumour** ***(images b, c, and d).***

PET/CT offers a unique opportunity to further improve the image quality of functional lung imaging owing to its superior sensitivity for detecting radioactive substances, higher spatial and temporal resolution and commercial availability of respiratory gated 4D acquisition systems [24]. By substituting the conventional ^99m^Tc radionuclide with ^68^Ga, a positron emitter, it is now possible to perform CT co-registered perfusion ^68^Ga-macroaggregated albumin (MAA) PET [25], Figure 1. We have previously reported that non-gated 3D V/Q PET/CT has superior image quality and provides fully tomographic images with potential for better regional quantitation of lung function as compared to V/Q SPECT/CT in the context of pulmonary embolism [26]. We have further improved this technique through the use of respiratory gated (4D) acquisition, which can reduce blurring caused by respiration motion and resultant artefact at the lung bases [27, 28]. We have also described methodology for deformable image registration in the context of Galligas ventilation PET and CT ventilation datasets. The use of 4D-V/Q PET imaging allows for fully quantitative assessment of regional injury during lung irradiation (Figure 2). We aim to use this novel imaging technique to inform radiotherapy planning firstly by adaptation of RT planning pre-treatment to respect the individual patient’s lung tolerance. This may enable the treatment of a subgroup of patients that would not be considered eligible for curative RT based on population-based risk estimates of entire lung. Secondly, we aim to simulate adaptation of RT planning during the treatment course in order to personalise RT delivery in response to individual lung injury.

## Methods/Design

**Figure 3:**
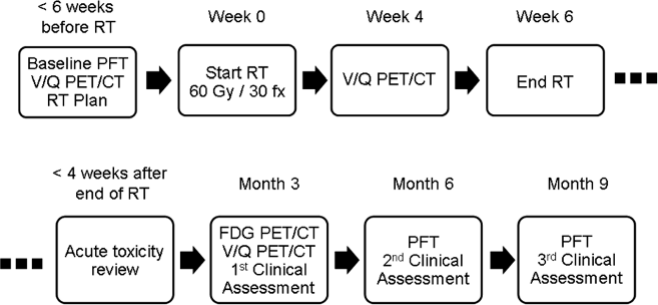
**Trial schema.**

This is a prospective single cohort observational study investigating *in-vivo* biomarkers radiation toxicity in *n* = 60 patients with NSCLC. The trial schema is demonstrated in Figure 3.

### Trial inclusion criteria

- Age ≥ 18 years;
- Written informed consent has been provided.
- FDG-PET scan performed for cancer staging
- Patients receiving curative intent radiotherapy for non-small cell lung cancer.
- Minimum dose of radiotherapy prescribed is 60Gy with or without chemotherapy
- ECOG performance status 0–2 inclusive

### Trial exclusion criteria

- Participant is not able to tolerate supine position on PET/CT bed for the duration of the PET/CT acquisitions, is not cooperative, or needs continuous nursing (e.g. patient from Intensive Care Unit).
- Pregnancy or breast-feeding
- Lung Spirometry: reversibility in FEV1 > 200mls and > 15% predicted change after bronchodilator

### Trial objectives

The primary objective of this study is to evaluate the pattern of regional pulmonary perfusion and ventilation as demonstrated on V/Q PET/CT before, during and after a course of radiotherapy in patients with NSCLC.

The secondary objectives are to:

1. Assess whether serial changes in global lung ventilation and perfusion as demonstrated on PFTs are related to regional lung ventilation and perfusion changes as demonstrated on V/Q PET/CT
2. Describe the quality of the co-registration of V/Q PET/CT with i) respiratory attenuated concurrent 4DCT or with ii) conventional CT alone.
3. Describe a dose/response relationship between delivered dose and ventilation/perfusion changes demonstrated on V/Q PET/CT
4. Whether ventilation/perfusion mismatches and lung density demonstrated on baseline V/Q PET/CT are correlated with baseline PFT parameters.
5. To simulate administration of biologically adapted radiotherapy techniques personalised to individual lung function based on information gained from V/Q PET/CT at baseline and at mid-treatment

The exploratory objectives are to assess:

1. Whether mid-treatment or post-treatment changes in pulmonary perfusion measured by V/Q PET/CT are associated with delayed development of inflammatory changes in the radiation field as determined by FDG PET/CT at 3 months post-treatment.
2. Whether mid-treatment or post-treatment changes in pulmonary perfusion measured by V/Q PET/CT are associated with delayed disease control in the radiation field at 3 months post-treatment as determined by metabolic response in tumour on FDG PET/CT.
3. Whether radiological post-treatment changes in perfusion, density or metabolism in either lung or tumour are associated with the development of clinical toxicity
4. Whether changes in regional ventilation and perfusion demonstrated on V/Q PET/CT is different between patients with and without radiological evidence of fibrosis.

### Biologically adaptive planning

Patients in this study will be treated with conventional radiotherapy, 60 Gy in 30 fractions over six weeks. Functionally adaptive planning tailored to ventilated and perfused lung volumes will be performed at 2 timepoints:

1. Simulated alternative plan based on baseline V/Q PET/CT functional volumes of lung
2. Simulated biologically adapted RT based on the *week-four interim* V/Q PET/CT information;
  For all patients: − an accelerated dose-schedule in the final week of RT onwards will be simulated, delivering 1.8Gy bi-daily to an isotoxic dose satisfying original Organ at Risk (OAR) constraints. Plans will be created using conventional anatomical methods using CT information alone.
  For patients deficits: − an accelerated dose-escalated schedule in the final week of RT onwards will be simulated, delivering 1.8Gy bi-daily to an isotoxic dose satisfying original Organ at Risk (OAR) constraints. Plans will be created using functional lung volumes dervied from the V/Q PET/CT.

Organ at Risk (OAR) dose measures for which limits will be set include: Lung: volume receiving 20 Gy, 30 Gy and mean dose, Spinal canal: maximum dose, Oesophagus: volume receiving 50 Gy, 60 Gy, mean and maximum dose, Heart: volume receiving 40 Gy, 60 Gy and mean dose.

For each model, we will record and analyse the following dose parameters:

- Tumour control probability (TCP) and normal tissue complication probability (NTCP)
- The maximum, mean and standard deviation of escalated dose achievable
- OAR doses at each dose increment and OAR preventing escalation to the next dose increment

### Statistical considerations

The proposed sample size was calculated based on the capacity to detect the rate of clinical pneumonitis in those patients not demonstrating perfusion deficits during radiotherapy. Based on initial findings, it is expected that 60% of patients will have no evidence of perfusion injury at the interim V/Q PET/CT scan. We anticipate that these patients to have an ~10% rate of clinical pneumonitis at 1 year, as compared with historical clinical pneumonitis rates of ~30% at 1 year for patients treated with curative intent RT. With a sample size of 60, a 3 year-accrual period, and a minimum of 1-year follow-up for toxicity assessment, then allowing for a 2-sided type I error rate of 0.05 the power of the study to distinguish between the two groups is equal to 80%.

## Translational substudy

Participation in a translational substudy will be offered for up to 45 patients of the total 60 patients recruited into this trial.

### Inflammatory cytokine release

Cytokine release in response to ionizing radiation is a documented phenomenon and may play a major role in subsequent radiation induced lung toxicity (reviewed in [29–33]. Fractionated radiation creates a constant complex stress response and a cytokine profile is different to that induced by a single radiation dose [34]. RT-related plasma concentrations of one or more cytokines in humans have correlated with lung toxicity. Transforming growth factor (TGF)-β1 [35–38], interleukin (IL)-6 and IL-10 [39, 40] during RT have been suggested as possible risk markers in these studies. However, other studies have reported contradictory or negative findings [41, 42]. In this study, we propose to analyse a partial selection of cytokines from a commercial human inflammatory cytokine panel of 22 cytokines. The rationale for the composition of 22 potential biomarkers for lung tissue toxicity is based on several published reports dissecting inflammatory and radiation response.

### Assessment of γ-H2AX signal as a biodosimeter

DNA is the most significant target of radiation exposure for survival and carcinogenesis. An early response of the cell to ionizing radiation-induced DNA damage is a phosphorylation of a histone protein H2AX, forming γ-H2AX [43]. Hundreds to thousands of γ-H2AX molecules surround one DSB to form a focus which functions to open the chromatin structure and to serve as a platform for the accumulation of many factors involved in the DDR [44]. These sites can be marked with anti-γ-H2AX antibodies with fluorescent “tags”. The number of foci per cell is proportional to the radiation dose and follows well-studied kinetics in normal tissues [45, 46]. The γ-H2AX assay is considered to be the most sensitive modern assay for DSB detection and response to radiation doses as low as 1 mGy. This sensitivity allows detection of radiotherapy-induced DNA damage *in situ* in human lymphocytes [47]. In addition, the assay has another important feature; it measures a change which occurs very quickly with the maximal response is at 30 minutes to 1 hour after irradiation. Dose-dependent responses and persistence of foci make γ-H2AX assay a good biodosimeter for exposure of humans to ionizing radiation during radiological diagnostics or therapeutic treatments [47–49]. The application of this assay in the case of homogeneous total body irradiation is straightforward and relies on the measurement of the average number of γH2AX foci per cell. An approach has also been suggested to apply γH2AX assay as a biodosimeter for partial body irradiation to evaluate the irradiated fraction of the blood volume and the dose received by that fraction [50]. The approach exploits such measures as the fraction of lymphocytes with γH2AX foci and the average number of γH2AX foci per cell in this fraction. In the proposed study we plan to analyse distributions of cells (lymphocytes) with respect to the number of γH2AX foci as a further development of this approach. We expect that the analysis of distributions will allow us to deconvolute irradiated and non-irradiated subpopulations of lymphocytes and to estimate the fraction and the dose for irradiated subpopulation.

### Assessment of the abscopal effect using γ-H2AX

A novel approach to assessment of an individual patient risk from lung radiotherapy is the assessment of ‘out-of-field’ radiation induced changes. The appearance of genome abnormalities and loss of viability in cells other than those directly hit with ionizing radiation (IR) is a well-documented process known as the radiation-induced bystander effect [51]. An important question is whether such effects demonstrated *in vitro* also exist *in vivo*. In classic radiobiology there is the so-called abscopal (out-of field or distant) effect, where irradiation of one organ results in a change in another, unirradiated organ [52]. Although possibly caused by scatter from the main radiation source, abscopal effects may also suggest the presence of bystander-like processes in whole organisms. Inflammatory mediators, such as chemokines, cytokines, and prostaglandins [53] as well as reactive oxygen and nitrogen species [54, 55] mediate this effect. DNA damage has been reported in noncancerous cells neighboring tumors [56, 57] for example, in normal liver tissue adjacent to hepatocellular carcinoma [58]. We hypothesize that the intensity of ‘out-of-field’ radiation induced changes demonstrated during and after a course of radiotherapy will predict for individual patient risk for developing lung radiation toxicity. The first step for ‘proof of principle’ is to document abscopal changes through detection of γ-H2AX foci within non-irradiated (bystander) tissues (hair follicles from the eyebrows of participating patients). These changes will be compared (when available) to changes in chest hairs from within the irradiated portal (to act as a ‘control’).

### Translational substudy methodology

Up to 45 patients enrolled into the study will be invited to participate in this translational substudy. In addition to the investigations mandated in the protocol, blood samples and hair follicles will be collected and processed at the following timepoints:

- At baseline before treatment (this will be taken at the time of blood collection prior to injection of the Ga-68 tracer for the baseline PET scan)
- 1-hour after the first fraction of radiotherapy
- No longer than 1 hour before the second fraction radiotherapy (approximately 24 hours after the first fraction of radiotherapy)
- Mid-treatment at 4 weeks. (this will be taken at the time of blood collection prior to injection of the Ga-68 tracer for the mid-treatment PET scan)
- 3-months post-treatment (this will be taken at the time of blood collection prior to injection of the Ga-68 tracer for the post-treatment PET scan)

To process the blood sample for biodosimetric analysis, the following methodology will be used:

- Collection of lymphocytes by Ficoll gradient separation.
- Fixing and immunofluorescent staining using a mouse γ-H2AX primary antibody (Abcam) and secondary anti-mouse antibody labelled with Alexa488 fluorescent dye (Millipore).
- Imaging with confocal microscopy and automatic analysis of γ-H2AX positive cells.

To process the blood samples for assessment of cytokine release, the following methodology will be used:

- Serum will be separated and frozen at −80°C until analysis.
- Analysis will be performed using a multiplex ELISA based platform

To process the hair follicles for assessment of bystander radiation effect, the following methodology will be used:

- 3 hair follicles will be plucked from the eyebrow region of participating patients at each time-point.
- The hairs will be fixed, immunostained for γ-H2AX, and processed for microscopy and analysis.

## Discussion

Lung cancer remains the leading cause of cancer death in Australia and RT is a primary treatment modality for the most common form, NSCLC. Current evidence suggests that the ideal dose is a uniform 60Gy prescribed over 6 weeks to the majority of patients [59]. The two major outcomes of this research will be the generation of biologically personalised RT plans adapted to individual patient lung tolerance, and data regarding clinically useful early biomarkers to predict for patient outcomes. 4D-^68^Ga-V/Q PET/CT represents a novel imaging biomarker for lung function and allows for highly accurate measurements of lung ventilation and perfusion. This clinical trial investigates the ability to biologically adapt RT in patients with NSCLC using a state-of-the-art combination of clinical, imaging and serum biomarker analyses in order to achieve the aims of our research, which are: a) individualising RT to maximise the probability of curing lung cancer, b) increase the number of patients who may be suitable for curative radiotherapy by planning radiotherapy delivery to avoid functional lung, c) determine models for targeting dose intensified radiation whilst sparing the important functioning lung surrounding the tumour and d) determining the proportion of patients who could receive intensified doses safely within the constraints of surrounding organs, and how high these intensified doses would be. Furthermore, the major implications of establishing interim prognostic markers during RT include: e) the validation of prognostic indices to predict clinical behaviour and assess toxicity risk and f) providing an insight into normal lung behaviour during RT, thereby presenting an opportunity to enhance patient management, including the delivery of individually adapted RT. At the successful completion of this trial we plan to advance this research by implementing a clinical trial of biologically adaptive radiotherapy that is personalised to both the patient’s pre-treatment regional lung function and observed functional lung injury during treatment.
